# Prokaryotic community profiles from Guánica state forest caves revealed by 16S rRNA gene amplicon sequencing

**DOI:** 10.1128/mra.00816-26

**Published:** 2026-10-12

**Authors:** Isabel Aguirre-Ortiz, Francisco J. Román-Soto, Camila M. Martínez-Llaurador, Peter Camacho-Nazario, Naomy M. Chaparro-Mata, Josué Rodríguez-Ramos, Ángel M. Nieves-Rivera, Miguel J. Martínez-González, Miguel Canals-Mora, Carlos Rios-Velazquez

**Affiliations:** 1 Microbial Biotechnology and Bioprospecting Laboratory, Biology Department, University of Puerto Rico, Mayagüez, Puerto Rico, USA; 2 Industrial Biotechnology Program, University of Puerto Rico, Mayagüez, Puerto Rico, USA; 3 Biological Sciences Division, Pacific Northwest National Laboratory 6865, Richland, Washington, USA; 4 Grupo Espeleológico del Oeste, Inc. (GEO), Mayagüez, Puerto Rico, USA; Indiana University Bloomington, Bloomington, Indiana, USA

**Keywords:** 16S rRNA gene, caves, Guánica Dry Forest, Puerto Rico, sequencing, prokaryotes

## Abstract

Cave environments harbor microorganisms adapted to extreme conditions. Prokaryotic diversity was assessed using 16S rRNA gene amplicon sequencing across four caves in the Guánica State Forest. The resulting data set characterizes phylum-level community composition within an understudied subtropical dry-forest cave system and provides baseline information for future microbial and ecological investigations.

## ANNOUNCEMENT

Caves are extreme environments characterized by darkness, high humidity, and limited nutrients (1). Despite their oligotrophic nature, they harbor microbial communities involved in biogeochemical cycling and the production of bioactive compounds (2–4). Culture-independent approaches provide broader insights into cave microbial diversity because many cave microorganisms are difficult to cultivate under laboratory conditions. Although caves in Puerto Rico’s northern limestone karst have recently been characterized using 16S rRNA gene and ITS amplicon sequencing, southern cave systems, particularly those in Guánica State Forest, remain microbiologically underexplored (5, 6). Unlike the more humid cave environments of northern Puerto Rico, Guánica’s caves occur within a subtropical dry forest characterized by lower rainfall and seasonal water availability, offering a distinct ecological setting (7, 8).

**Fig 1:**
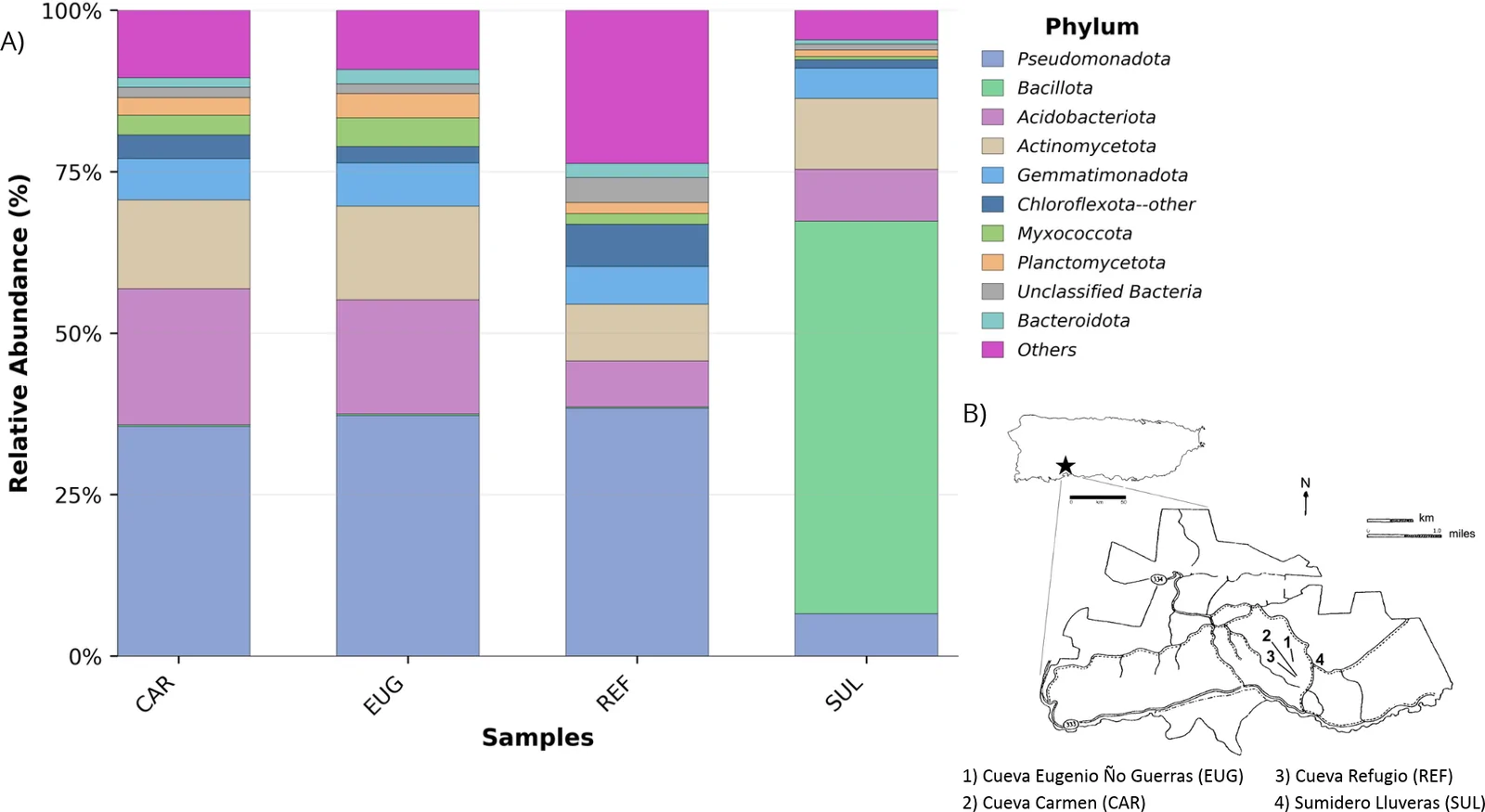
Relative abundance of prokaryotic communities at the phylum level across four caves in the Guánica State Forest. (**A**) Taxa bar plots showing the relative abundance of prokaryotic phyla across samples; phyla representing <5% were grouped as “others.” (**B**) Map of Guánica State Forest indicating the locations of the sampled caves. The map was generated by Ángel Nieves-Rivera.

In this study, four caves from Guánica State Forest were sampled (Table 1). Thirty grams of superficial bat guano-enriched soil were collected from each cave in sterile Whirl-Pak bags and stored at −20°C for 1 week before DNA extraction. Genomic DNA was extracted from 10 g of each sample suspended in 20 mL Z-buffer (100 mM Tris-HCl [pH 8.0], 100 mM sodium phosphate, 100 mM EDTA [pH 8.0], 1.5M NaCl, and 1% cetyltrimethylammonium bromide) containing 20 mg lysozyme (≥780,000 U). Cells were disrupted by repeated freeze–thaw cycles, followed by lysis with 2.25 mL sodium dodecyl sulfate and 1.5 mL guanidinium isothiocyanate at 65°C for 2 h. Lysates were centrifuged at 4,000× *g* for 10 min and extracted with an equal volume of chloroform. The aqueous phase was precipitated with one volume of isopropanol at −20°C overnight. DNA pellets were centrifuged at 4,000× *g* for 45 min, air-dried, resuspended in T_10_E_10_ (10 mM Tris-HCl [pH 8.0], 10 mM EDTA), and stored at −20°C (9–12). DNA fragments up to 25 kb were purified by gel electrophoresis followed by β-Agarase I treatment (13). Purified DNA was submitted to Molecular Research DNA Laboratory (MR DNA, Shallowater, TX, USA) for bTEFAP Illumina 20K sequencing targeting the V3–V4 region of the 16S rRNA gene. Amplification used primers 341F (5′-CCTACGGGNGGCWGCAG-3′) and 785R (5′-GACTACHVGGGTATCTAATCC-3′) (14) with the HotStarTaq Plus Master Mix Kit (Qiagen, USA). PCR consisted of 95°C for 5 min; 30 cycles of 95°C for 30 s, 53°C for 40 s, and 72°C for 1 min; and 72°C for 10 min. Sequencing was performed on an Illumina MiSeq instrument using 2 × 300 bp paired-end reads. Demultiplexed reads were processed using QIIME 2 version 2026.1 within a Python 3.10.14 environment (15). Forward and reverse primers were removed using Cutadapt before DADA2 denoising (16). Based on read-quality profiles, both forward and reverse reads were truncated at 230 bp with no additional 5′ trimming; consensus chimera removal was used. Of 2,205,068 input reads, 1,989,901 passed filtering, 1,231,742 merged, and 702,968 were retained as non-chimeric reads. Read loss occurred mainly during paired-end merging and chimera removal. Phylum-level analysis showed that *Pseudomonadota* was the most abundant group (29.41%), followed by *Bacillota* (15.39%), *Acidobacteriota* (13.47%), *Actinomycetota* (11.99%), *Gemmatimonadota* (5.92%), *Chloroflexota* (3.49%), *Myxococcota* (2.41%), and *Planctomycetota* (2.30%) (Fig. 1).

**TABLE 1:** Cave localization and sampling dates

Sampled caves	Sample ID	Coordinates	Collection date	Input reads	Percentage of input passed filter	Denoised	Percentage of non-chimeric reads
Cueva Carmen	CAR	17° 57′ 33″ N, 66° 51′10″ W	28/06/2025	552,203	90.12	462,858	32.48
Cueva Eugenio (Ño) Guerras	EUG	17° 57′ 43″ N, 66° 50′50″ W	13/10/2025	560,268	88.94	460,934	31.03
Cueva El Refugio	REF	17° 57′ 35″ N, 66° 51′11″ W	28/06/2025	507,398	91.32	436,541	40.17
Sumidero Lluveras	SUL	17° 57′ 51″ N, 66° 50′45″ W	13/10/2025	585,199	90.68	517,671	24.94

## Data Availability

The raw data from this project have been deposited in the National Center for Biotechnology Information under the BioProject accession number PRJNA1457879. The samples were distributed as follows: Cueva Carmen (CAR) (SRA accession number SRX33109045), Cueva Eugenio No Guerra (EUG) (SRA accession number SRX33109046), Cueva El Refugio (REF) (SRA accession number SRX33109047), Sumidero Lluveras (SUL) (SRA accession number SRX33109048). The BioProject also includes five previously published cave sequences that are not part of the data set reported in this study.
